# Evaluation of Total Antioxidant Activity and Phenolic Profiles of Calabrian Breba Figs: A Detailed Study of Pulp and Skin from 29 *Ficus carica* L. Accessions

**DOI:** 10.3390/foods13244035

**Published:** 2024-12-13

**Authors:** Alessandra De Bruno, Rocco Mafrica, Valentino Branca, Amalia Piscopo, Marco Poiana

**Affiliations:** 1 Department of Human Sciences and Promotion of the Quality of Life, San Raffaele University, 00166 Rome, Italy; alessandra.debruno@uniroma5.it; 2 Department of AGRARIA, University Mediterranea of Reggio Calabria, Vito, 89124 Reggio Calabria, Italy; valentino.branca@unirc.it (V.B.); amalia.piscopo@unirc.it (A.P.); mpoiana@unirc.it (M.P.)

**Keywords:** ABTS assay, antioxidant activity, bioactive components, brebas, DPPH assay, figs, flavonoids, phenolic profile

## Abstract

This work was carried out on twenty-nine fig accessions cultivated in the Calabria region (Italy). The main antioxidant parameters were determined with the aim of selecting superior genotypes and supporting the establishment of new commercial orchards specializing in breba production. The studied samples were divided into two main classes characterized by different skin fruit colors (light and dark). The total antioxidant capacity (DPPH and ABTS), total polyphenols, and total flavonoids of the fig accessions were analyzed spectrophotometrically, while the individual phenolic components were identified and quantified by UHPLC-PDA. The phenolic profiles showed significant differences among the tested samples and between flesh and skin. The highest concentrations of bioactive components were found in the skin rather than the flesh. The total polyphenol contents varied between 15 and 50 mg of gallic acid equivalents (GAE) per 100 g of fresh weight (FW) in the pulp and between 18 and 251 mg GAE per 100 g (FW) in the skin.

## 1. Introduction

Figs (*Ficus carica* L.) belong to the Moraceae family and are flavorful infructescences that develop on fig trees [1]. Fig fruits are consumed worldwide, but more than 70% of global production is concentrated in Mediterranean countries [2]. Italy is one of the major fig-producing countries, with production primarily concentrated in the southern regions, such as Calabria, where large cultivation areas exist and there is a very rich and diversified autochthonous fig germplasm in terms of genetic aspects [3], tree morphological and functional characteristics [4], phenological traits [5], and fruit pomological and qualitative characteristics [6].

Based on their bloom and maturity stage, Ficus fruits are classified as brebas and/or figs. Brebas are dormant figs that do not begin their development until the following spring. They are larger and juicier and are normally eaten fresh, while figs appear from the bloom of the year. These are smaller and can be eaten both fresh and dried [7] or used to make jam [8]. Ripe edible figs have a thick skin and a sweet flesh containing small seeds, which are usually unnoticeable but may provide a slight crunch when chewed [9]. A fresh fig can be eaten either with or without its skin [10]. The skin color of fig varieties can vary from green to black-violet, depending on the specific pigment compounds present [11]. Figs are rich in essential nutrients, including dietary fibers, amino acids, vitamins, minerals, sugars, organic acids, carotenoids, and antioxidant polyphenols (primarily flavonoids and phenolic acids). Due to these beneficial properties, figs have been used in traditional medicine for centuries, particularly for their health-promoting effects in the treatment of gastrointestinal, respiratory, inflammatory, metabolic, and cardiovascular disorders; for this reason, figs are considered a good source of bioactive compounds [12,13,14]. Furthermore, figs’ nutritional importance extends beyond basic nutrients.

Scientific research has proven that a diet based on fruit and vegetables has beneficial effects on human health. In particular, the intake of vegetable foods with bright colors is very important for the prevention of several diseases, for example, atherosclerosis, cardiac disorders, and cancer, because they are rich in bioactive compounds such as polyphenols and carotenoids [15]. Fig fruits have a high antioxidant capacity and provide many benefits for human health, such as anti-inflammatory and antidiabetic properties and antibacterial activities [16,17]. The bioactive profile of fig fruits depends on many factors and is characterized by a wide range of phenolics that vary depending on cultivar and varietal type and on other factors such as soil and fruit maturity [10,18].

Figs contain two primary types of phytochemicals: polyphenols and carotenoids [12]. Among phenolic compounds, phenolic acids and flavonoids (flavanols, flavonols, and anthocyanidins) have been identified, and, in addition, different agronomic parameters, such as fruit variety and harvest season, can influence the antioxidant profiles of the fruits [17]. Quercetin rutinoside is one of the major individual phenolics [12,18].

An additional differentiation for fig trees is the partition into “biferous” trees, which produce two crops of figs per year—the first crop during the spring season (late May and the end of June) and the second crop in the summer season (mid-July and early September) [19]—and “uniferous” trees, which produce a single crop of figs [7].

The idea for this work stems from a previous study conducted by Mafrica et al. [6], where forty fig accessions were analyzed for qualitative parameters. Of these forty, twenty-nine accessions were selected for the evaluation of antioxidant parameters. In particular, twenty-nine biferous and uniferous accessions of fig fruits (light- to dark-skinned) were studied for the evaluation of total polyphenols, total flavonoids, DPPH, ABTS, and individual phenolic compounds. To achieve these research objectives, the following methodology was employed.

## 2. Materials and Methods

### 2.1. Reagents and Standards

To measure total antioxidant activity, 2,2′-azino-bis acid (3-ethylbenzothia-zolin-6-sulfonic acid) (ABTS), 2,2-diphenyl-1-picrylhydrazyl (DPPH), Trolox, and Folin–Ciocalteu’s phenol reagent were purchased from Sigma Chemical Co. (St. Louis, MO, USA). Ultrapure water, methanol, and acetonitrile (UHPLC-MS-grade; Carlo Erba, Italy) were used for chromatographic analysis. The following standards were used for the quantification of phenolic compounds: catechin, epicatechin, chlorogenic acid, quercetin, and rutin (Merck, Darmstadt, Germany).

### 2.2. Sample Collection and Preparation

#### 2.2.1. Fruit Collection

Twenty-nine different Calabrian breba accessions (*Ficus carica* L.) were collected from commercial and experimental orchards which were of a similar age and shared structural characteristics and were situated at elevations ranging from 80 to 200 m above sea level. The orchards’ characteristics were reported in another work of ours [6]. The breba fruits belonged to biferous and uniferous accessions characterized by different skin colors (light and dark). Fruits were harvested during the crop season of 2019 at the commercial ripening stage, identified by the characteristic color of the variety and the ability to apply slight finger pressure to the fruits. The fruits were collected from various positions around the canopy at a height of 160 cm (for a total of 30 fruits per accession).

#### 2.2.2. Storage Conditions

Immediately after harvesting, the fruits were placed in refrigerated containers at 3 °C and promptly transported to the laboratory, where they were analyzed for key antioxidant parameters within 48 h.

#### 2.2.3. Sample Preparation

After selecting the fig samples based on different skin colors, they were manually peeled and the epicarps were separated from the pulp. Subsequently, both parts—pulp (P) and epicarp (E)—were ground separately using a laboratory blender and subjected to extraction for subsequent analysis for the evaluation of the antioxidant capacity.

### 2.3. Physical Analysis

#### 2.3.1. Color Measurement

Color was measured at four points on each fruit: two opposite points near the pedicel and two opposite points near the ostiole, representing the earliest and latest areas of color change, respectively. Measurements were taken from a total of 10 fruits per sample. Color analyses were performed with a colorimeter (model: CR-300; Minolta, Osaka, Japan). Color was evaluated according to the CIELab method by measuring the parameters L*, a*, and b*. a* and b* values were used to compute the intensity of color, namely, the chroma value: (a2 + b2)1/2.

#### 2.3.2. Classification Criteria

**Figure 1:**
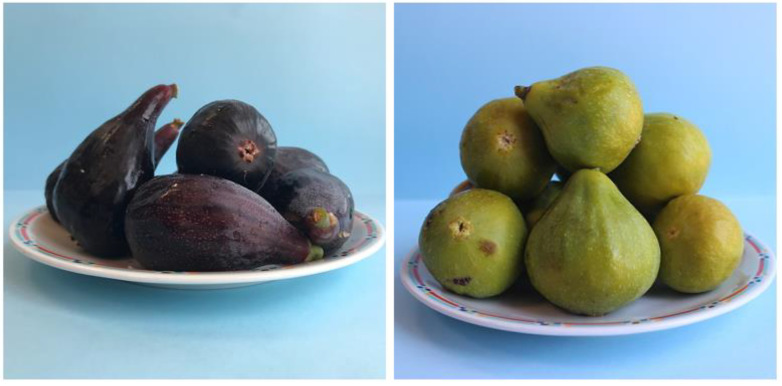
Examples of analyzed fig samples: Bifera nera *CS148* (dark skin) and Unifera Bianca *CS166* (light skin).

On the basis of the color of the skin, the fig samples (example Figure 1) were divided into two main groups and sub-groups, and the denominations are reported in Table 1.

### 2.4. Chemical Analysis

#### 2.4.1. Extraction Procedure

The extraction procedure used to determine the antioxidant properties of fig fruits was carried out following the method previously described by Ercisli et al. [20]. Samples of 10 g (P and E) were extracted with 50 mL of buffer (HCl/methanol/water, 2:80:18, *v*/*v*/*v*). After, the solution was centrifuged at 10,000 rpm for 5 min in a refrigerated centrifuge (NF 1200R; Nüve, Ankara, Turkey) and the liquid fraction was recovered and filtered.

The obtained extracts were used for the following analyses:

#### 2.4.2. Total Phenolic Contents (TPCs)

TPCs were analyzed spectrophotometrically using the modified Folin–Ciocalteu colorimetric method described by Singleton et al. [21]. A quantity of 100 µL of extract (P-E) was reacted in a volumetric flask (5 mL) with 2000 µL of Folin–Ciocalteu reagent (diluted 1:10), 2000 µL of Na_2_CO_3_, and 900 µL of distilled water. The whole was left to react for 2 h in the dark at 25 °C, then subjected to spectrophotometric measurement at 760 nm. The TPCs were determined by comparing the absorbance of each extract with a standard response curve generated using gallic acid. The results are expressed as mg of gallic acid equivalents per 100 g (mg GAE 100 g^−1^) of sample (FW).

#### 2.4.3. Total Flavonoid Contents (TFCs)

TFCs were determined colorimetrically as described previously by Zhishen et al. [22]. A quantity of 0.5 mL of extract (P-E) was mixed with 5 mL of distilled water and 0.3 mL of sodium nitrite (NaNO_2_, diluted 1:20); after 5 min, 3 mL of AlCl_3_ (diluted 1:10) was added, and after 6 min, 2 mL of NaOH (1 M) was added. Everything was brought to volume with distilled water. The mixture was shaken well, and the measurement was performed at 510 nm using a spectrophotometer. The TFCs were determined using a (+)-catechin standard curve and expressed as the mean mg of (+)-catechin equivalents per 100 g (mg CE 100 g^−1^) of fruit (FW).

#### 2.4.4. Total Antioxidant Assays

DPPH (1,1-diphenyl-2-picrylhydrazyl) and ABTS (2,20-azino-bis acid (3-ethylbenzothiazolin-6-sulfonic acid) assays were applied for the determination of total antioxidant capacity, following the methodologies reported by Romeo et al. [23]. The total antioxidant activity was expressed as the percentage of inhibition for the DPPH assay, calculated by applying the following formula:

% Inhibition = 100 × (At_0_ − At_end_)/At_0_

where At_0_ represents the absorbance of the DPPH_solution at the initial time and At_end_ is the absorbance measured after 30 min. For the ABTS assays, the results were expressed as mmol Trolox equivalents 100 g (mmol TE 100 g^−1^) of the sample (FW).

#### 2.4.5. Individual Phenolic Compounds (IPCs)

IPCs were analyzed by ultra-high-performance liquid chromatography (UHPLC), following the chromatographic conditions described by Romeo et al. [23]. Five microliters of extract (filtered with a 0.22 μm pore size membrane filter, RC) were injected into a UHPLC PLATINblue (Knauer, Berlin, Germany), and chromatograms were recorded at different wavelengths (280, 320, 360, and 510 nm). Compound identification was performed by comparing their retention times and UV spectra with those of pure commercial standards (concentrations ranging from 1 to 100 mg/L). Regression equations (REs), Pearson correlation coefficients (r), and limits of detection (LODs) and quantification (LOQs) for each antioxidant compound are reported in Table 2. The results are expressed as mg per 100 g (mg 100 g^−1^) of sample (FW).

### 2.5. Statistical Analysis

The results obtained in this experimentation were reported as mean values ± standard deviations (SDs) of three measurements.

#### 2.5.1. ANOVA Details

Significant differences (*p* < 0.05) among samples were determined using one-way analysis of variance followed by Tukey’s post hoc test.

#### 2.5.2. Correlation Test

Pearson’s correlation test was used to calculate the correlation coefficients (r) between TPCs, TFCs, and the antioxidant assays (DPPH and ABTS).

#### 2.5.3. Classical Cluster Methodology

The analysis was conducted using SPSS Software (Version 15.0), based on the statistical analysis of TPC from the 29 fig accessions, using the distance measure and the ‘complete linkage method’.

#### 2.5.4. Software Used

Data processing was performed with SPSS Software (Version 15.0; SPSS Inc., Chicago, IL, USA).

## 3. Results and Discussion

Based on the determination of the color parameters (L*, a*, b*, and chroma) of the figs’ skins, the twenty-nine fig accessions were divided into two main groups (Table 3): light-skinned (LS) and dark-skinned (DS) figs. The skin color of brebas is one of the most important factors that influences consumer acceptability, as it is useful for assessing the status of ripening in fruits [24,25]. The obtained results revealed in this work showed great statistical variability (*p* < 0.01) in the color of breba fruits, from light green to dark purple. Considering the high variability of the samples, we subdivided the samples into subgroups: two for LS, namely, yellow-green-skinned (YS) and light-green-skinned (SLG); and two for DS, namely, purple-skinned (PS) and dark-purple-skinned (DPS). For LS, the chromatic parameters ranged between L* 35.93 and 62.62, a* −3.85 and 12.05, b* 20.40 and 47.74, and C* 22.24 and 47.92, while for DS, they ranged between L* 21.48 and 37.48, a* 2.09 and 16.00, b* −0.36 and 9.66, and C* 2.31 and 19.12. The results obtained in this work fall within the ranges reported by other authors, such as Ercisli et al. [20]. Chroma is one of the most important parameters used to describe the quality of food and has a considerable influence on acceptance by consumers [26].

The chemical and morphological characteristics of breba fruits were reported in another of our papers [5,6].

The phytochemical composition of fig fruits is often affected by the cultivar, but also by other factors, such as the color, the part of the fruit, and the level of maturity [27]. The antioxidant compounds and the phenolic profiles in particular that characterize the Breba accessions are very important for determining the quality of the final products in order to promote their consumption as fresh products, in addition to being useful for nutritional and health purposes. The phenolic profile mainly affects parameters such as the flavor and odor of the fruit [7]. Breba fruits contain high amounts of polyphenols and flavonoids and at the same time exhibit higher antioxidant activity. Moreover, as reported by Viuda-Martos et al. [28], the total amount of phenolic compounds (phenolic acids, flavonoids, and anthocyanins) present in fig fruits could be considered an important parameter for revealing their antioxidant capacity with the aim of promoting the fruits as a natural source of antioxidants.

**Figure 2:**
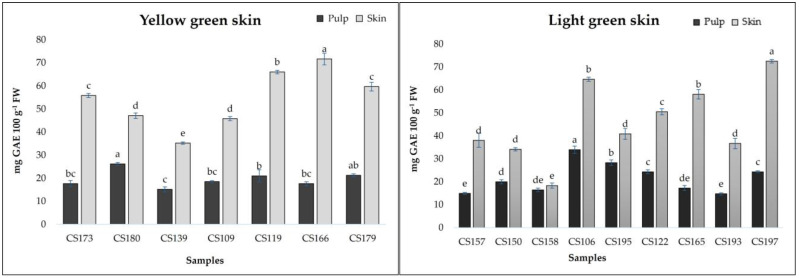
Total phenolic compounds of pulp and skin in “light-skinned breba accessions”. The data are presented as means. Different letters indicate significant differences at *p* < 0.05, as determined by Tukey’s post hoc test.

**Figure 3:**
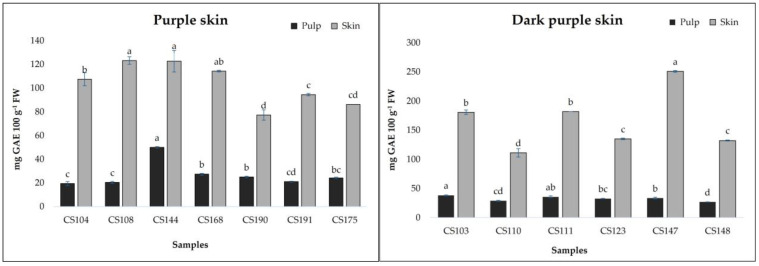
Total phenolic compounds in pulp and skin of “dark-skinned fig accessions”. The data are presented as means. Different letters indicate significant differences at *p* < 0.05, as determined by Tukey’s post hoc test.

Figure 2 and Figure 3 show the results related to total phenolic contents measured in breba fruits. TPCs ranged from 14.72 to 49.92 mg GAE 100 g^−1^ FW (pulp) and from 18.30 to 251.81 mg GAE 100 g^−1^ FW (skin). The obtained results are comparable to those obtained by Vallejo et al. [14] for the skin of fresh figs (19.1 mg/100 g for to 140.2 mg/100 g FW) and by Ercisli et al. [20] (237 mg/100 g GAE FW). The analyzed fig samples revealed statistical differences (*p* < 0.01) not only among the different typologies of breba fruit (LS and DS), but also within the subgroups (YS, LGS, PS, and DPS). As can be seen in Figure 2 and Figure 3, it is clear that the skin showed the highest TPCs, with a major concentration in the sample *CS147* (250.81 mg GAE 100 g^−1^ FW); however, almost all the “bifera nera” samples exhibited high TPCs in their skin (>100 mg GAE 100 g^−1^ FW). On the other hand, even the pulp showed good TPC values, ranging from 14.72 (*CS193*) to 49.92 in *CS144*, with higher results for “bifera nera”. Also, other authors found that fig fruit with dark skin showed higher TPC values [20,29,30]. Building upon these findings, we next examined the individual phenolic compounds.

**Figure 4:**
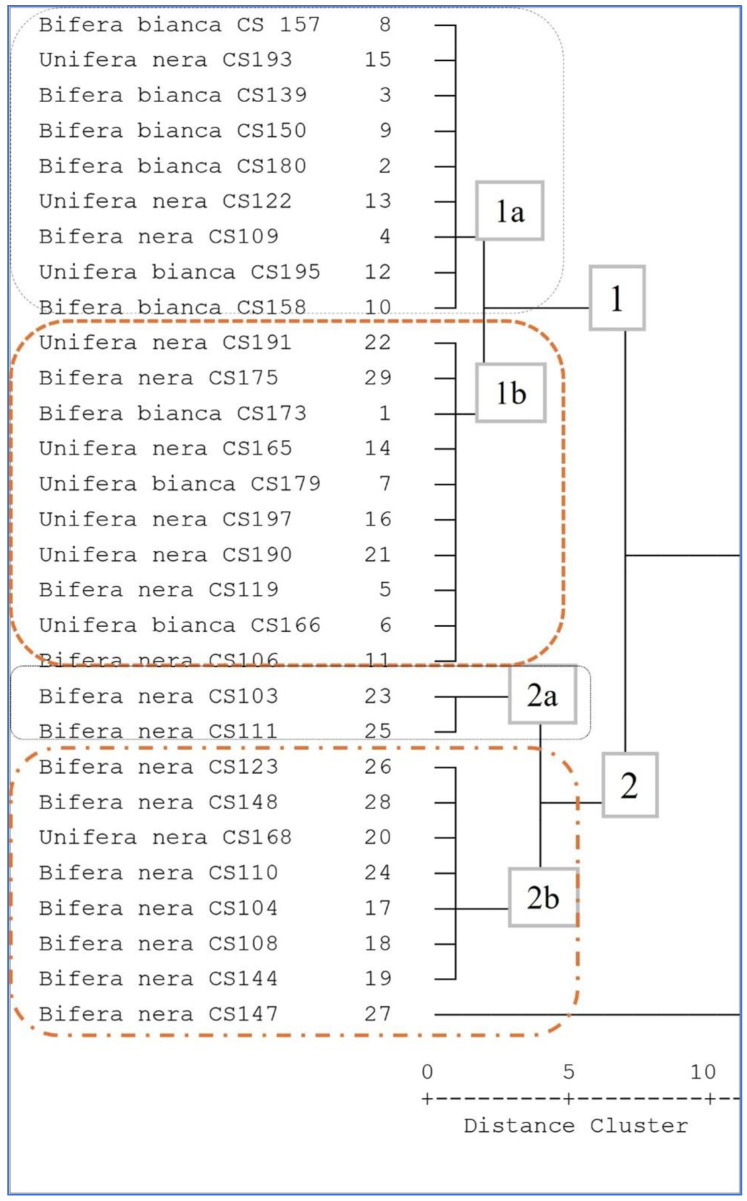
Cluster analysis based on the statistical analysis of 29 morphological traits of fig accessions, based on the distance and the ‘complete linkage method’. Group 1 includes 19 accessions with a lower TPC content (1a: 9 accessions with the lowest TPC values; 1b: 10 Accessions with slightly higher TPC levels than 1a); Group 2 includes 10 accessions with a higher TPC content (2a: 2 accessions with the highest level of TPC; 2b: 8 Accessions with high level of TPC, but lower than 2a).

Cluster analysis highlighted the variation among the fig samples in terms of total phenolic contents. Indeed, Figure 4 shows the obtained dendrogram, in which the fig fruits are divided into two groups with similar total phenolic contents.

The cluster analysis (Figure 4) classified the fig accessions into two main groups (Group 1 and Group 2) based on total polyphenol content (TPC) and other distinguishing characteristics, such as skin color. Each group was further divided into subgroups, reflecting finer differences among the accessions.

Group 1 comprised a total of 19 accessions characterized by a lower TPC. This group predominantly included figs with lighter skin tones and featured both uniferous and biferous types. It was further divided into two subgroups.

Subgroup 1a included nine accessions with the lowest TPC values recorded in the entire dataset.

Subgroup 1b comprised 10 accessions with slightly higher TPC levels than those in Subgroup 1a but whose levels were still lower than those in Group 2. This subgroup exhibited greater diversity in skin color, ranging from light to intermediate tones, and included a mix of uniferous and biferous types.

Group 2 consisted of 10 accessions characterized by higher TPCs. This group was distinctly marked by a predominance of dark-skinned figs, a trait visually and biochemically associated with a higher polyphenol content. This group was also subdivided into two subgroups:

Subgroup 2a consisted of two specific accessions (Bifera Nera *CS103* and *CS111*), which exhibited the highest TPC values among all the analyzed accessions.

Subgroup 2b included eight accessions, primarily of the Bifera Nera type, with a single exception (Unifera Nera *CS168*). These accessions exhibited high TPC levels but lower levels than those observed in Subgroup 2a. The limited variability within this subgroup reflects small differences in TPC content that may be attributed to genetic or environmental factors.

The cluster analysis results highlight a clear separation of fig accessions based on polyphenol content and morphological/visual traits, such as skin color and cropping type (uniferous vs. biferous).

Total flavonoid contents (TFCs) were measured spectrophotometrically, and the results are reported in Table 4. Flavonoids represent a subgroup of phenolic compounds in breba fruit accessions, and their presence was confirmed with the TFC/TPC ratio values. In general, the highest TFC/TPC ratios were shown in the skin of the samples with values of about 0.17–0.39 in LS and 0.22–0.45 in DS. Significant statistical differences (*p* < 0.01) were found among all samples, between LS and DS and between pulp and skin. Most flavonoid compounds are present in the fruit skin, the values for which ranged between 5.81 and 26.25 in LS and between 20.08 and 65.26 mg CE 100 g^−1^ FW in DS accessions. Thus, the highest TFC results were shown in the subgroup of DPS (*CS147* sample). Instead, the highest TFC content in fig pulp was evidenced in the samples *CS144* (15.30 mg CE 100 g^−1^ FW) and *CS179* (12.22 mg CE 100 g^−1^ FW). The TFC data obtained in this study are similar and sometimes higher than the data reported by other authors [30,31,32].

The antioxidant activity of different breba fruits (pulp and skin) was quantified by two methods: DPPH and ABTS assays (Table 5 and Table 6), and we found significant differences among the accessions (*p* < 0.01).

In agreement with the above data, total antioxidant activity revealed higher values in DS accessions than in LS accessions. Regarding the determinations carried out for the “light-skinned breba accessions”, the results are shown in Table 5. The DPPH assay results ranged between 2.06% (*CS173*) and 10.02% (*CS158*) for pulp and from 6% (*CS157*) to 17.99% (*CS166*) for the skin of brebas. The results obtained for “dark-skinned breba accessions” (DS) are reported in Table 5. The DPPH assay results ranged from 3.91% (*CS104*) to 9.18% (*CS147*) in the pulp and from 13.88% (*CS190*) to 52.95% (*CS147*) in the skin of brebas.

In general, accessions with dark skin revealed higher total antioxidant capacities than those with light skin, and the sample that showed the highest antioxidant capacity in the DPPH assay was *CS147* (Black Biferous), both in the flesh and the skin. The findings of this study support previous reports, confirming that figs with a dark skin exhibit higher antioxidant capacity compared to white figs [33,34].

For the ABTS assay, the samples that showed higher results were *CS166* for LS pulp and *CS173* for skin (Table 5), while for DS, *CS144* showed the highest value for pulp and *CS147* showed the highest value for skin (Table 6). The latter sample (*CS147*) showed higher antioxidant activity in both assays.

Pearson correlation analysis revealed significant relationships between total phenolic content and antioxidant activity assays (DPPH and ABTS), with notable differences between the skin and the pulp of the fig samples.

For the skin, TPC showed a very high correlation with the DPPH assay, with coefficients ranging from r = 0.838 (LGS) to r = 0.947 (DPS). This highlights the skin’s substantial contribution to antioxidant capacity, driven by its phenolic content.

In the pulp, a stronger correlation was observed between TPC and the ABTS assay, with the PS sample showing the highest coefficient.

Correlations between total flavonoid content and antioxidant activity were weaker, with exceptions in DPS pulp (r = 0.760) and LGS skin (0.814), indicating a lesser but still relevant contribution of flavonoids.

The most representative individual phenolic compounds present in the studied breba fruits are shown in Table 7. A total of five phenolic compounds were detected, which belong to different chemical classes, such as phenolic acids (chlorogenic acid), flavonoids (catechin and epicatechin), and flavonols (quercetin and rutin). These compounds, as reported by other authors, are among the most representative bioactive compounds present in the peel, flesh, and whole fruits of figs [35]. The identification and quantification of these phenolic compounds through UHPLC analysis confirmed the results obtained from spectrophotometric determinations, providing robust evidence of their presence and distribution in the breba samples. Higher concentrations of individual phenolic compounds were found in the breba fruit samples, with statistical differences among the samples (*p* < 0.01). The two most prevalent compounds determined were catechin and rutin, followed by the other compounds, epicatechin, chlorogenic acid, and quercetin. Regarding the LS samples, rutin was the most abundant compound present in the skin samples and ranged between 0.98 and 38.88 mg 100 g^−1^ FW, while catechin contents were high both in flesh and in skin (ranging between 1.37 and 35.52 and between 2.60 and 30.77 mg 100 g^−1^ FW, respectively). Meanwhile, the DS breba accessions showed the highest contents of catechin in the skin of the fruits, with values that ranged between 54.87 and 597.81 mg 100 g^−1^ FW.

Concerning the LS fig fruits, a higher content of catechin was highlighted in the *CS195* sample, prevailing in the pulp (35.52 mg 100 g^−1^ FW); moreover, among the light-skinned samples, this variety of fruit is the richest in individual phenolic compounds, while the *CS158* sample showed a lower amount of individual phenolic compounds, both in the pulp and in the skin. Rutin contents were higher in the LS samples compared with the DS samples, while catechin contents were higher in the dark figs, particularly in the skin, with values of 448.37 mg 100 g^−1^ FW. The data obtained for the characterization of individual phenolic compounds confirmed the results reported by several other authors, namely, that the skin contains higher concentrations of phenolic compounds compared to the pulp [17,36,37] and that fruit color also influences the concentration and composition of these antioxidant active compounds [29,38].

## 4. Conclusions

In this study, the antioxidant properties of some fig accessions cultivated in the Calabrian region were assessed for their nutritional aspects. The antioxidant attributes displayed by fruits are crucial for various reasons, one of which is that intake of natural foods can serve as a source of antioxidants, i.e., functional natural foods. The findings of this study underscore significant variation among figs concerning their antioxidant properties and their demonstration of high concentrations of polyphenols and flavonoids. Particularly noteworthy are the accessions characterized by a darker skin color. All assessed antioxidant parameters exhibited higher levels in the skin, emphasizing the importance of consuming the whole fruit in fresh consumption. The fruit can also be subject to various processing treatments, e.g., in the production of jams and dried fruits.

Regarding the pulp, it is rich in flavonoids, particularly catechin, which is very important for human health due to its prevention of various diseases. Therefore, the consumption of fresh figs could be a valid alternative for the intake of natural antioxidants.

## Figures and Tables

**Table 1:** Denominations of breba fruits and classification based on skin color.

Accession	Fruit Skin Color	Accession	Fruit Skin Color
Light-Skinned Breba Fruits		Dark-Skinned Breba Fruits	
*Bifera bianca CS173*	Yellowish	*Bifera nera CS104*	Purple
*Bifera bianca CS180*	Yellowish	*Bifera nera CS108*	Purple
*Bifera bianca CS139*	Yellow-green	*Bifera nera CS144*	Purple
*Bifera nera CS109*	Yellow-green	*Unifera nera CS168*	Purple
*Bifera nera CS119*	Yellow-green	*Unifera nera CS190*	Purple
*Unifera bianca CS166*	Yellow-green	*Unifera nera CS191*	Purple
*Unifera bianca CS179*	Yellow-green	*Bifera nera CS175*	Purple
*Bifera bianca CS157*	Light green	*Bifera nera CS103*	Dark purple
*Bifera bianca CS150*	Light green	*Bifera nera CS110*	Dark purple
*Bifera bianca CS158*	Light green	*Bifera nera CS111*	Dark purple
*Bifera nera CS106*	Light green	*Bifera nera CS123*	Dark purple
*Unifera bianca CS195*	Light green	*Bifera nera CS147*	Dark purple
*Unifera nera CS122*	Light green	*Bifera nera CS148*	Dark purple
*Unifera nera CS165*	Light green		
*Unifera nera CS193*	Light green		
*Unifera nera CS197*	Light green		

**Table 2:** Method development through UHPLC-PDA.

Compounds	RE	r	LOD (µg g^−1^)	LOQ (µg g^−1^)
Catechin	y = 6.22x − 34.04	0.999	0.011	0.02
Epicatechin	y = 6.95x + 11.32	0.999	0.035	0.03
Chlorogenic acid	y = 52.15x − 100.73	0.999	0.053	0.03
Quercetin	y = 59.09x + 119.01	0.999	0.046	0.02
Rutin	y = 46.07x − 14.44	0.999	0.045	0.04

**Table 3:** Denominations of breba fruits and classification based on skin color.

	Accession	L*	a*	b*	C*
Light-Skinned Breba Fruits	*Bifera bianca CS173*	57.84 ^a^	1.65 ^bcd^	47.74 ^a^	47.92 ^a^
	*Bifera bianca CS180*	57.47 ^ab^	3.17 ^bc^	45.66 ^a^	45.97 ^a^
	*Bifera bianca CS139*	54.29 ^ab^	−2.65 ^d^	35.00 ^b^	35.13 ^b^
	*Bifera nera CS109*	52.78 ^bc^	9.18 ^a^	44.23 ^a^	45.36 ^a^
	*Bifera nera CS119*	42.15 ^d^	3.11 ^b^	32.36 ^b^	32.73 ^b^
	*Unifera bianca CS166*	48.40 ^c^	0.84 ^bcd^	42.48 ^a^	42.67 ^a^
	*Unifera bianca CS179*	53.98 ^ab^	−2.05 ^cd^	33.90 ^b^	34.00 ^b^
	Sign.	**	**	**	**
	*Bifera bianca CS157*	52.24 ^b^	5.92 ^bc^	29.90 ^ab^	30.52 ^ab^
	*Bifera bianca CS150*	49.67 ^b^	4.60 ^cd^	26.60 ^bc^	27.10 ^bcde^
	*Bifera bianca CS158*	62.62 ^a^	−3.85 ^e^	28.47 ^abc^	28.90 ^bcd^
	*Bifera nera CS106*	39.59 ^c^	11.25 ^ab^	23.78 ^cd^	26.74 ^bcde^
	*Unifera bianca CS195*	49.8 ^b^	−0.16 ^de^	25.05 ^bcd^	25.06 ^cde^
	*Unifera nera CS122*	39.18 ^c^	7.52 ^abc^	20.46 ^d^	22.24 ^e^
	*Unifera nera CS165*	35.93 ^c^	12.05 ^a^	20.40 ^d^	24.11 ^de^
	*Unifera nera CS193*	40.88 ^c^	8.13 ^abc^	33.51 ^a^	34.94 ^a^
	*Unifera nera CS197*	39.38 ^c^	11.83 ^a^	27.58 ^bc^	30.32 ^abc^
	Sign.	**	**	**	**
Dark-Skinned Breba Fruits	*Bifera nera CS104*	23.50 ^c^	11.34 ^ab^	6.43 ^ab^	13.12 ^ab^
	*Bifera nera CS108*	27.68 ^b^	5.87 ^bc^	8.61 ^ab^	10.96 ^b^
	*Bifera nera CS144*	37.48 ^a^	3.72 ^c^	8.81 ^ab^	9.66 ^b^
	*Unifera nera CS168*	23.18 ^c^	13.20 ^a^	9.66 ^a^	16.92 ^ab^
	*Unifera nera CS190*	26.12 ^bc^	12.48 ^ab^	7.46 ^ab^	14.69 ^ab^
	*Unifera nera CS191*	29.41 ^b^	16.00 ^a^	9.13 ^ab^	19.12 ^a^
	*Bifera nera CS175*	25.46 ^bc^	10.78 ^abc^	4.48 ^b^	11.88 ^b^
	Sign.	**	**	*	**
	*Bifera nera CS103*	34.97 ^a^	3.15	0.84	3.28
	*Bifera nera CS110*	25.50 ^b^	8.04	2.74	8.91
	*Bifera nera CS111*	24.43 ^b^	3.76	−0.26	4.26
	*Bifera nera CS123*	21.75 ^b^	3.06	0.77	3.21
	*Bifera nera CS147*	21.48 ^b^	2.09	−0.36	2.31
	*Bifera nera CS148*	22.16 ^b^	6.61	2.08	7.03
	Sign.	**	ns	ns	ns

The data are presented as means. ** Significance at *p* < 0.01; * Significance at *p* < 0.05; ns: not significant. Different letters indicate significant differences at *p* < 0.05, as determined by Tukey’s post hoc test.

**Table 4:** Total flavonoids of different “breba accessions”.

Light-Skinned Breba Accessions					Dark-Skinned Breba Accessions				
YS					PS				
Samples	Pulp	TF/TPC	Skin	TF/TPC	Samples	Pulp	TF/TPC	Skin	TF/TPC
*CS173*	1.04 ± 0.05 ^d^	0.06	21.98 ± 0.20 ^b^	0.39	*CS104*	1.71 ± 0.03 ^f^	0.09	47.99 ± 0.14 ^a^	0.45
*CS180*	4.46 ± 0.06 ^b^	0.17	12.68 ± 0.19 ^e^	0.27	*CS108*	2.03 ± 0.10 ^f^	0.10	27.72 ± 0.81 ^d^	0.22
*CS139*	4.14 ± 0.19 ^b^	0.27	9.26 ± 0.50 ^f^	0.26	*CS144*	15.30 ± 0.05 ^a^	0.31	45.50 ± 0.09 ^b^	0.37
*CS109*	2.54 ± 0.14 ^c^	0.14	12.99 ± 0.24 ^e^	0.28	*CS168*	7.37 ± 0.29 ^b^	0.27	36.02 ± 0.09 ^c^	0.32
*CS119*	2.96 ± 0.26 ^c^	0.14	15.68 ± 0.14 ^d^	0.24	*CS190*	3.09 ± 0.01 ^e^	0.12	20.08 ± 0.05 ^f^	0.26
*CS166*	2.50 ± 0.04 ^c^	0.15	26.25 ± 0.74 ^a^	0.37	*CS191*	6.34 ± 0.20 ^c^	0.30	25.03 ± 0.25 ^e^	0.27
*CS179*	12.22 ± 0.10 ^a^	0.58	20.07 ± 0.01 ^c^	0.34	*CS175*	4.85 ± 0.16 ^d^	0.20	23.92 ± 0.44 ^e^	0.28
Sign.	**		**		Sign.	**		**	
**LGS**					**DPS**				
**Samples**	**Pulp**	**TF/TPC**	**Skin**	**TF/TPC**	**Samples**	**Pulp**	**TF/TPC**	**Skin**	**TF/TPC**
*CS157*	5.82 ± 0.05 ^b^	0.39	12.78 ± 0.10 ^d^	0.34	*CS103*	9.13 ± 0.00 ^a^	0.24	43.82 ± 0.19 ^c^	0.24
*CS150*	4.43 ± 0.00 ^d^	0.22	10.13 ± 0.33 ^e^	0.30	*CS110*	5.51 ± 0.09 ^c^	0.19	42.23 ± 0.24 ^c^	0.38
*CS158*	4.37 ± 0.04 ^d^	0.27	5.81 ± 0.13 ^g^	0.32	*CS111*	5.86 ± 0.19 ^bc^	0.17	50.03 ± 3.20 ^b^	0.28
*CS106*	3.14 ± 0.04 ^e^	0.09	16.79 ± 0.24 ^b^	0.26	*CS123*	3.00 ± 0.13 ^d^	0.09	45.99 ± 0.19 ^bc^	0.34
*CS195*	7.75 ± 0.04 ^a^	0.27	9.07 ± 0.04 ^f^	0.22	*CS147*	4.68 ± 0.06 ^c^	0.14	65.26 ± 0.64 ^a^	0.26
*CS122*	4.65 ± 0.10 ^c^	0.19	8.51 ± 0.39 ^f^	0.17	*CS148*	6.85 ± 0.77 ^b^	0.26	43.28 ^c^	0.33
*CS165*	1.47 ± 0.06 ^f^	0.09	13.74 ± 0.19 ^c^	0.24	Sign.	**		**	
*CS193*	1.40 ± 0.08 ^f^	0.09	8.67 ± 0.09 ^f^	0.24					
*CS197*	4.71 ± 0.02 ^c^	0.19	24.10 ± 0.34 ^a^	0.33					
Sign.	**		**						

The data are presented as means (mg of CE 100 g^−1^). ** Significance at *p* < 0.01. Different letters indicate significant differences at *p* < 0.05, as determined by Tukey’s post-hoc test.

**Table 5:** Total antioxidant parameters of different “light-skinned breba accessions” (YS and LGS).

	DPPH (% Inactivation)		ABTS (mmol TE 100 g^−1^)	
YS Samples	Pulp	Skin	Pulp	Skin
*CS173*	2.06 ± 0.02 ^d^	10.54 ± 1.20 ^bc^	1.00 ± 0.07 ^de^	10.04 ± 0.34 ^a^
*CS180*	9.08 ± 0.14 ^a^	9.70 ± 0.79 ^bc^	1.64 ± 0.04 ^cd^	1.90 ± 0.03 ^d^
*CS139*	3.98 ± 0.09 ^c^	6.62 ± 0.16 ^c^	2.38 ± 0.19 ^b^	3.12 ± 0.15 ^c^
*CS109*	4.48 ± 0.29 ^c^	9.48 ± 1.80 ^bc^	1.84 ± 0.03 ^bc^	2.64 ± 0.05 ^cd^
*CS119*	7.06 ± 0.16 ^b^	17.68 ± 1.32 ^a^	2.34 ± 0.07 ^b^	4.79 ± 0.34 ^b^
*CS166*	7.15 ± 0.44 ^b^	17.99 ± 0.34 ^a^	4.42 ± 0.40 ^a^	5.20 ± 0.16 ^b^
*CS179*	4.44 ± 0.13 ^c^	11.33 ± 0.66 ^b^	0.83 ± 0.05 ^e^	4.54 ± 0.10 ^b^
Sign.	**	**	**	**
LGS Samples	Pulp	Skin	Pulp	Skin
*CS157*	3.65 ± 0.25 ^cd^	6.00 ± 0.60 ^g^	1.25 ± 0.05 ^f^	2.53 ± 0.50 ^bc^
*CS150*	6.18 ± 0.25 ^b^	9.89 ± 0.28 ^de^	1.55 ± 0.08 ^ef^	1.66 ± 0.04 ^cd^
*CS158*	10.02 ± 0.03 ^a^	6.53 ± 0.43 ^fg^	1.41 ± 0.11 ^f^	1.51 ± 0.01 ^d^
*CS106*	9.10 ± 0.53 ^a^	11.42 ± 0.53 ^bcd^	2.95 ± 0.05 ^b^	3.85 ± 0.04 ^a^
*CS195*	5.61 ± 0.13 ^b^	10.34 ± 0.96 ^cde^	3.79 ± 0.09 ^a^	2.86 ± 0.07 ^b^
*CS122*	4.38 ± 0.24 ^c^	13.00 ± 0.40 ^ab^	1.53 ± 0.02 ^f^	2.88 ± 0.21 ^b^
*CS165*	9.29 ± 0.32 ^a^	12.21 ± 0.32 ^bc^	1.94 ± 0.06 ^de^	2.92 ± 0.25 ^b^
*CS193*	3.32 ± 0.07 ^d^	8.38 ± 0.35 ^ef^	1.99 ± 0.13 ^d^	2.89 ± 0.12 ^b^
*CS197*	4.30 ± 0.09 ^cd^	14.31 ± 0.51 ^a^	2.47 ± 0.20 ^c^	4.27 ± 0.24 ^a^
Sign.	**	**	**	**

The data are presented as means. ** Significance at *p* < 0.01. Different letters indicate significant differences at *p* < 0.05, as determined by Tukey’s post hoc test.

**Table 6:** Total antioxidant parameters of different “dark-skinned breba accessions” (PS and DPS).

	DPPH (% Inactivation)		ABTS (mmol TE 100 g^−1^)	
PS Samples	Pulp	Skin	Pulp	Skin
*CS104*	3.91 ± 0.26 ^d^	28.63 ± 0.09 ^a^	2.08 ± 0.03 ^c^d	9.14 ± 0.48 ^b^
*CS108*	8.91 ± 0.35 ^a^	27.93 ± 1.74 ^a^	2.71 ± 0.14 ^b^	11.12 ± 0.24 ^a^
*CS144*	4.19 ± 0.03 ^cd^	30.96 ± 1.62 ^a^	5.64 ± 0.29 ^a^	11.43 ± 0.13 ^a^
*CS168*	5.02 ± 0.14 ^bc^	22.87 ± 1.95 ^b^	2.17 ± 0.14 ^cd^	7.92 ± 0.24 ^c^
*CS190*	5.13 ± 0.49 ^bc^	13.88 ± 0.73 ^c^	2.18 ± 0.03 ^bcd^	5.08 ± 0.17 ^e^
*CS191*	4.66 ± 0.16 ^cd^	20.96 ± 0.02 ^b^	1.86 ± 0.06 ^d^	7.49 ± 0.10 ^c^
*CS175*	5.80 ± 0.06 ^b^	18.20 ± 0.17 ^bc^	2.55 ± 0.02 ^bc^	6.24 ± 0.10 ^d^
Sign.	**	**	**	**
DPS Samples	Pulp	Skin	Pulp	Skin
*CS103*	7.03 ± 0.39 ^b^	43.62 ± 1.60 ^b^	3.45 ± 0.00 ^b^	14.07 ± 0.32 ^d^
*CS110*	4.52 ± 0.06 ^c^	24.93 ± 1.19 ^d^	2.86 ± 0.12 ^c^	8.93 ± 0.15 ^e^
*CS111*	8.64 ± 0.20 ^a^	42.41 ± 1.20 ^b^	4.99 ± 0.08 ^a^	17.41 ± 0.60 ^c^
*CS123*	4.66 ± 0.30 ^cd^	27.25 ± 1.18 ^d^	2.00 ± 0.09 ^d^	19.46 ± 0.14 ^b^
*CS147*	9.18 ± 0.27 ^a^	52.95 ± 0.81 ^a^	5.04 ± 0.05 ^a^	21.44 ± 0.20 ^a^
*CS148*	7.55 ± 0.05 ^b^	35.80 ± 1.93 ^c^	1.33 ± 0.04 ^e^	13.28 ± 0.27 ^d^
Sign.	**	**	**	**

The data are presented as means. ** Significance at *p* < 0.01. Different letters indicate significant differences at *p* < 0.05, as determined by Tukey’s post hoc test.

**Table 7:** Individual phenolic compounds in LS breba fruit accessions (mg 100 g^−1^).

Light-Skinned Breba Accessions										
IPC	Catechin		Epicatechin		Chlorogenic A.		Quercetin		Rutin	
Samples	Pulp	Skin	Pulp	Skin	Pulp	Skin	Pulp	Skin	Pulp	Skin
*CS173*	1.87 ± 0.01 ^f^	3.33 ± 0.00 ^b^	0 ^f^	n.d.	0.33 ± 0.01 ^f^	0.69 ± 0.00 ^e^	n.d.	0.02 ± 0.00 ^e^	0 ^e^	17.85 ± 0.40 ^d^
*CS180*	5.22 ± 0.01 ^b^	4.27 ± 0.02 ^b^	2.01 ± 0.01 ^b^	n.d.	0.44 ± 0.02 ^c^	0.76 ± 0.00 ^d^	n.d.	0.56 ± 0.01 ^d^	1.55 ± 0.03 ^a^	11.53 ± 0.03 ^f^
*CS139*	5.76 ± 0.02 ^a^	5.76 ± 0.02 ^b^	1.59 ± 0.04 ^c^	n.d.	0.36 ± 0.00 ^e^	1.22 ± 0.00 ^b^	n.d.	0.03 ± 0.00 ^e^	0.09 ± 0.00 ^d^	22.47 ± 0.08 ^c^
*CS109*	3.560.05 ^e^	6.48 ± 1.11 ^b^	2.38 ± 0.01 ^a^	n.d.	0.61 ± 0.00 ^b^	0.51 ± 0.00 ^f^	n.d.	1.18 ± 0.00 ^c^	0.32 ± 0.01 ^b^	14.90 ± 0.20 ^e^
*CS119*	4.70 ± 0.14 ^c^	7.24 ± 0.06 ^b^	0.85 ± 0.09 ^e^	n.d.	0.32 ± 0.01 ^f^	0.89 ± 0.00 ^c^	n.d.	4.36 ± 0.01 ^a^	0.04 ± 0.02 ^e^	22.96 ± 0.02 ^c^
*CS166*	1.96 ± 0.01 ^f^	13.40 ± 0.69 ^a^	0.12 ± 0.02 ^f^	n.d.	0.65 ± 0.00 ^a^	3.47 ± 0.00 ^a^	n.d.	1.89 ± 0.03 ^b^	0 ^e^	39.88 ± 0.58 ^a^
*CS179*	3.92 ± 0.01 ^d^	7.18 ± 0.02 ^b^	1.40 ± 0.02 ^d^	n.d.	0.42 ± 0.00 ^d^	0.76 ± 0.00 ^d^	n.d.	0.02 ± 0.00 ^e^	0.17 ± 0.00 ^c^	36.63 ± 0.04 ^b^
Sign.	**	**	**		**	**		**	**	**
*CS157*	3.33 ± 0.01 ^d^	3.50 ± 0.02 ^f^	0.20 ± 0.01 ^cd^	n.d.	0.50 ± 0.00 ^b^	0.28 ± 0.00 ^i^	n.d.	0.60 ± 0.01 ^e^	0.39 ± 0.00 ^c^	9.52 ± 0.03 ^e^
*CS150*	3.48 ± 0.45 ^d^	3.14 ± 0.01 ^g^	1.75 ± 0.01 ^a^	n.d.	0.40 ± 0.00 ^d^	0.47 ± 0.00 ^f^	n.d.	0.24 ± 0.00 ^g^	0 ^f^	3.79 ± 0.02 ^f^
*CS158*	1.79 ± 0.01 ^f^	2.60 ± 0.02 ^h^	0.21 ± 0.01 c^d^	n.d.	0.41 ± 0.00 ^d^	0.39 ± 0.00 ^g^	n.d.	0.27 ± 0.00 ^g^	0.12 ± 0.00 ^e^	0.98 ± 0.01 ^g^
*CS106*	18.65 ± 0.05 ^b^	20.20 ± 0.16 ^b^	1.55 ± 0.01 ^a^	n.d.	0.45 ± 0.00 ^b^	1.17 ± 0.00 ^c^	n.d.	0.88 ± 0.00 ^d^	0.12 ± 0.00 ^e^	13.97 ± 0.04 ^d^
*CS195*	35.52 ± 0.04 ^a^	13.79 ± 0.08 ^c^	0.11 ± 0.01 ^d^	n.d.	2.03 ± 0.00 ^a^	2.67 ± 0.00 ^a^	n.d.	0.52 ± 0.00 ^f^	0.81 ± 0.01 ^a^	30.43 ± 0.39 ^a^
*CS122*	4.94 ± 0.02 ^c^	10.36 ± 0.01 ^e^	1.68 ± 0.01 ^a^	n.d.	0.32 ± 0.00 ^e^	0.34 ± 0.00 ^h^	n.d.	0.63 ± 0.01 ^e^	0.19 ± 0.00 ^d^	8.30 ± 0.04 ^e^
*CS165*	3.66 ± 0.01 ^d^	30.77 ± 0.06 ^a^	0.40 ± 0.02 ^bc^	n.d.	0.43 ± 0.00 ^c^	0.69 ± 0.00 ^e^	n.d.	1.70 ± 0.01 ^b^	0 ^f^	19.92 ± 0.63 ^c^
*CS193*	1.37 ± 0.01 ^f^	11.71 ± 0.03 ^d^	0 ^d^	n.d.	0.27 ± 0.00 ^f^	0.89 ± 0.00 ^d^	n.d.	1.03 ± 0.03 ^c^	0 ^f^	9.33 ± 0.04 ^de^
*CS197*	2.52 ± 0.02 ^e^	13.56 ± 0.01 ^c^	0.52 ± 0.05 ^b^	n.d.	0.31 ± 0.00 ^e^	1.55 ± 0.00 ^b^	n.d.	2.19 ± 0.02 ^a^	0.46 ± 0.01 ^b^	28.36 ± 0.26 ^b^
Sign.	**	**	**		**	**		**	**	**
**Dark-Skinned Breba Accessions**										
**IPC**	**Catechin**		**Epicatechin**		**Chlorogenic A.**		**Quercetin**		**Rutin**	
**Samples**	**Pulp**	**Skin**	**Pulp**	**Skin**	**Pulp**	**Skin**	**Pulp**	**Skin**	**Pulp**	**Skin**
*CS104*	5.23 ± 0.00 ^f^	113.87 ± 0.81 ^d^	1.25 ± 0.02 ^d^	n.d.	0.37 ± 0.00 ^e^	0.02 ± 0.00 ^f^	n.d.	3.12 ± 0.02 ^b^	0.04 ± 0.00 ^f^	24.86 ± 0.02 ^c^
*CS108*	4.39 ± 0.01 ^g^	194.72 ± 1.74 ^b^	2.09 ± 0.01 ^b^	n.d.	1.30 ± 0.00 ^a^	1.47 ± 0.00 ^d^	n.d.	6.38 ± 0.01 ^a^	0 ^f^	26.37 ± 0.02 ^b^
*CS144*	14.21 ± 0.14 ^a^	139.40 ± 0.14 ^c^	2.93 ± 0.08 ^a^	n.d.	0.45 ± 0.01 ^b^	2.08 ± 0.00 ^c^	n.d.	0.32 ± 0.00 ^g^	1.01 ± 0.04 ^c^	20.02 ± 0.10 ^d^
*CS168*	13.27 ± 0.04 ^b^	71.37 ± 1.74 ^e^	0.54 ± 0.02 ^e^	n.d.	0.42 ± 0.00 ^c^	2.49 ± 0.00 ^b^	n.d.	1.79 ± 0.01 ^c^	2.38 ± 0.02 ^a^	27.44 ± 0.06 ^a^
*CS190*	8.35 ± 0.01 ^d^	54.87 ± 0.88 ^f^	1.84 ± 0.02 ^c^	n.d.	0.36 ± 0.00 ^e^	1.11 ± 0.00 ^e^	n.d.	1.05 ± 0.01 ^e^	0.28 ± 0.00 ^d^	17.88 ± 0.11 ^e^
*CS191*	7.45 ± 0.02 ^e^	238.88 ± 1.81 ^a^	0.12 ± 0.02 ^f^	n.d.	0.32 ± 0.01 ^f^	4.73 ± 0.00 ^a^	n.d.	0.97 ± 0.01 ^f^	0.13 ± 0.00 ^e^	14.34 ± 0.12 ^f^
*CS175*	9.84 ± 0.00 ^c^	115.96 ± 0.77 ^d^	0.67 ± 0.01 ^e^	n.d.	0.39 ± 0.00 ^d^	2.06 ± 0.00 ^c^	n.d.	1.53 ± 0.01 ^d^	1.10 ± 0.00 ^b^	24.85 ± 0.25 ^c^
Sign.	**	**	**		**	**		**	**	**
*CS103*	32.53 ± 0.00 ^a^	288.57 ± 0.02 ^c^	5.31 ± 0.06 ^a^	n.d.	0.66 ± 0.00 ^d^	2.14 ± 0.00 ^e^	n.d.	0.22 ± 0.00 ^e^	1.34 ± 0.03 ^a^	32.96 ± 0.02 ^a^
*CS110*	11.47 ± 0.06 ^d^	257.17 ± 1.08 ^d^	0.96 ± 0.01 ^e^	n.d.	0.59 ± 0.00 ^e^	5.05 ± 0.07 ^d^	n.d.	1.16 ± 0.01 ^cd^	0.15 ± 0.00 ^d^	13.92 ± 0.11 ^f^
*CS111*	28.52 ± 0.01 ^b^	448.37 ± 18.55 ^b^	4.49 ± 0.01 ^c^	n.d.	1.60 ± 0.00 ^b^	10.90 ± 0.03 ^b^	n.d.	20.79 ± 0.30 ^a^	1.13 ± 0.01 ^b^	23.60 ± 0.15 ^c^
*CS123*	11.02 ± 0.03 ^e^	261.96 ± 0.09 ^cd^	1.78 ± 0.00 ^d^	n.d.	0.41 ± 0.00 ^f^	5.15 ± 0.27 ^d^	n.d.	1.06 ± 0.01 ^d^	0.34 ± 0.00 ^c^	19.49 ± 0.02 ^d^
*CS147*	23.31 ± 0.06 ^c^	597.81 ± 2.63 ^a^	4.92 ± 0.11 ^b^	n.d.	1.79 ± 0.02 ^a^	16.69 ± 0.04 ^a^	n.d.	2.42 ± 0.01 ^b^	1.32 ± 0.04 ^a^	25.57 ± 0.33 ^b^
*CS148*	10.14 ± 0.00 ^f^	291.90 ± 2.00 ^c^	1.14 ± 0.01 ^e^	n.d.	0.98 ± 0.00 ^c^	7.06 ± 0.19 ^c^	n.d.	1.65 ± 0.01 ^c^	0.11 ± 0.00 ^d^	17.43 ± 0.32 ^e^
Sign.	**	**	**		**	**		**	**	**

The data are presented as means. ** Significance at *p* < 0.01; n.d.: not detected. Different letters indicate significant differences at *p* < 0.05, as determined by Tukey’s post hoc test.

## Data Availability

The original contributions presented in the study are included in the article; further inquiries can be directed to the corresponding author.

## References

[1] Bachir Bey M., Meziant L., Benchikh J., Louailech H. (2014). Deployment of response surface methodology to optimize recovery of dark fresh fig (*Ficus carica* L., var. *Azenjar*) total phenolic compounds and antioxidant activity. Food Chem..

[2] FAOSTAT (2020). FAO Database. https://faostat.fao.org.

[3] Costa F., Di Vaio C., Ferrara G., Fretto S., Mafrica R., Marchese A., Quartararo A., Marra F.P., Mennone C., Caruso T. (2017). Genetic diversity of fig (*Ficus carica* L.) genotypes grown in Southern Italy reveled by the use of SSR markers. Acta Hort..

[4] Caruso T., Mafrica R., Bruno M., Vescio R., Sorgonà A. (2021). Root architectural traits of rooted cuttings of two fig cultivars: Treatments with arbuscular mycorrhizal fungi formulation. Sci. Hortic..

[5] Mafrica R., De Bruno A., Piscopo A., Poiana M., Bruno M., Caruso T. (2021). Cultivar and accessions of fig (*Ficus carica* L.) for breba production selected within the autochthonous germplasm of Calabria (South Italy). Acta Hortic..

[6] Mafrica R., De Bruno A., Piscopo A., Poiana M. (2023). Performance evaluation of 40 fig accessions cultivated in Calabria: Study of qualitative parameters of breba production. J. Saudi Soc. Agric. Sci..

[7] Núñez-Gómez D., Legua P., Martínez-Nicolás J.J., Melgarejo P. (2021). Breba Fruits Characterization from Four Varieties (*Ficus carica* L.) with Important Commercial Interest in Spain. Foods.

[8] Mawa S., Husain K., Jantan I. (2013). *Ficus carica* L. (Moraceae): Phytochemistry, traditional uses and biological activities. Evid.-Based Complement. Altern. Med..

[9] Stover E., Aradhya M., Ferguson L., Crisosto C.H. (2007). The Fig: Overview of an Ancient Fruit. HortScience.

[10] Walia A., Kumar N., Singh R., Kumar V., Kaushik R., Kumar A.P. (2022). Bioactive compounds in *Ficus* fruits, their bioactivities, and associated health benefits: A review. J. Food Qual..

[11] Li J., An Y., Wang L. (2020). Transcriptomic Analysis of *Ficus carica* Peels with a Focus on the Key Genes for Anthocyanin Biosynthesis. Int. J. Mol. Sci..

[12] Sandhu A.K., Islam M., Edirisinghe I., Burton-Freeman B. (2023). Phytochemical Composition and Health Benefits of Figs (Fresh and Dried): A Review of Literature from 2000 to 2022. Nutrients.

[13] Rasool I.F., Aziz A., Khalid W., Koraqi H., Siddiqui S.A., AL-Farga A., Lai W.-F., Ali A. (2023). Industrial Application and Health Prospective of Fig (*Ficus carica*) By-Products. Molecules.

[14] Gaaliche B., Ladhari A., Zarrelli A., Mimoun M.B. (2019). Impact of foliar potassium fertilization on biochemical composition and antioxidant activity of fig (*Ficus carica* L.). Sci. Hortic..

[15] Ganoza-Yupanqui M.L., Muñoz-Acevedo A., Ybañez-Julca R.O., Mantilla-Rodríguez E., Zavala E., Gajardo S., Ríos M., Benites J., Martínez J.L. (2021). Potential antioxidant effect of fruit peels for human use by 5 different methods. Bol. Latinoam. Caribe Plant. Med. Aromat..

[16] Francini A., Sodini M., Vicario G., Raffaelli A., Gucci R., Caruso G., Sebastiani L. (2021). Cations and Phenolic Compounds Concentrations in Fruits of Fig Plants Exposed to Moderate Levels of Salinity. Antioxidants.

[17] Vallejo F., Marín J.G., Tomás-Barberán F.A. (2012). Phenolic compound content of fresh and dried figs (*Ficus carica* L.). Food Chem..

[18] Wojdyło A., Nowicka P., Carbonell-Barrachina A.A., Hernández F. (2016). Phenolic compounds, antioxidant and antidiabetic activity of different cultivars of *Ficus carica* L. fruits. J. Funct. Foods.

[19] Crisosto C.H., Bremer V., Ferguson L., Crisosto G.M. (2010). Evaluating quality attributes of four fresh figs (*Ficus carica* L.) cultivars harvested at two maturity stages. Hort. Sci..

[20] Ercisli S., Tosun M., Karlidag H., Dzubur A., Hadziabulic S., Aliman J. (2012). Color and Antioxidant Characteristics of Some Fresh Fig (*Ficus carica* L.) Genotypes from Northeastern Turkey. Plant Foods Hum. Nutr..

[21] Singleton V.L., Orthofer R., Lamuela-Raventós R.M. (1999). Analysis of total phenols and other oxidation substrates and antioxidants by means of folin-ciocalteu reagent. Methods Enzymol..

[22] Zhishen J., Mengcheng T., Jianming W. (1999). The determination of flavonoid contents in mulberry and their scavenging effects on superoxide radicals. Food Chem..

[23] Romeo R., De Bruno A., Imeneo V., Piscopo A., Poiana M. (2019). Evaluation of enrichment with antioxidants from olive oil mill wastes in hydrophilic model system. J. Food Process. Preserv..

[24] Pereira C., López Corrales M., Martín A., del Carmen Villalobos M., de Guía Córdoba M., Serradilla M.J. (2017). Physicochemical and Nutritional Characterization of Brebas for Fresh Consumption from Nine Fig Varieties (*Ficus carica* L.) Grown in Extremadura (Spain). J. Food Qual..

[25] Tsantili E. (1990). Changes during development of ‘Tsapela’ fig fruits. Sci. Hortic..

[26] Hendry G.A., Houghton J.D. (1996). Natural Food Colorants.

[27] Arvaniti O.S., Samaras Y., Gatidou G., Thomaidis N.S., Stasinakis A.S. (2019). Review on fresh and dried figs: Chemical analysis and occurrence of phytochemical compounds, antioxidant capacity and health effects. Food Res. Int..

[28] Viuda-Martos M., Sendra E., Sayas E., Pérez-Alvarez J.A., Fernández-López J. (2015). Fig (*Ficus carica*) Liquid Co-Products as New Potential Functional Ingredient: Physico-Chemical and In Vitro Antioxidant Properties. Nat. Prod. Commun..

[29] Del Caro A., Piga A. (2008). Polyphenol composition of peel and pulp of two Italian fresh fig fruits cultivars (*Ficus carica* L.). Eur. Food Res. Technol..

[30] Kamiloglu S., Capanoglu E. (2015). Polyphenol Content in Figs (*Ficus carica* L.): Effect of Sun-Drying. Int. J. Food Prop..

[31] Marinova D., Ribarova F., Atanassova M. (2005). Total phenolics and total flavonoids in Bulgarian fruits and vegetables. J. Univ. Chem. Technol. Metall..

[32] Bucic-Kojic A., Planinic M., Tomas S., Jokic S., Mujic I., Bilic M., Velic D. (2011). Effect of extraction conditions on the extractability of phenolic compounds from lyophilized fig fruits (*Ficus carica* L.). Pol. J. Food Nutr. Sci..

[33] Hssaini L., Charafi J., Hanine H., Ennahli S., Mekaoui A., Mamouni A., Razouk R. (2019). Comparative Analysis and PhysioBiochemical Screening of an Ex-Situ Fig (*Ficus carica* L.) Collection. Hortic. Environ. Biotechnol..

[34] Khadhraoui M., Bagues M., Artés F., Ferchichi A. (2019). Phytochemical Content, Antioxidant Potential, and Fatty Acid Composition of Dried Tunisian Fig (*Ficus carica* L.) Cultivars. J. Appl. Bot. Food Qual..

[35] Teruel-Andreu C., Andreu-Coll L., López-Lluch D., Sendra E., Hernández F., Cano-Lamadrid M. (2021). *Ficus carica* Fruits, By-Products and Based Products as Potential Sources of Bioactive Compounds: A Review. Agronomy.

[36] Piga A., del Caro A., Milella G., Pinna I., Vacca V., Schirru S. (2008). HPLC Analysis of Polyphenols in Peel and Pulp of Fresh Figs. Acta Hortic..

[37] Russo F., Caporaso N., Paduano A., Sacchi R. (2014). Phenolic Compounds in Fresh and Dried Figs from Cilento (Italy), by Considering Breba Crop and Full Crop, in Comparison to Turkish and Greek Dried Figs. J. Food Sci..

[38] Veberic R., Colaric M., Stampar F. (2008). Phenolic Acids and Flavonoids of Fig Fruit (*Ficus carica* L.) in the Northern Mediterranean Region. Food Chem..

